# Flaviviruses: Innate Immunity, Inflammasome Activation, Inflammatory Cell Death, and Cytokines

**DOI:** 10.3389/fimmu.2022.829433

**Published:** 2022-01-28

**Authors:** Yuhong Pan, Wenjun Cai, Anchun Cheng, Mingshu Wang, Zhongqiong Yin, Renyong Jia

**Affiliations:** ^1^ Research Center of Avian Disease, College of Veterinary Medicine, Sichuan Agricultural University, Chengdu, China; ^2^ Institute of Preventive Veterinary Medicine, Sichuan Agricultural University, Chengdu, China; ^3^ Key Laboratory of Animal Disease and Human Health of Sichuan Province, College of Veterinary Medicine, Sichuan Agricultural University, Chengdu, China

**Keywords:** innate immune, inflammasome, inflammatory cell death, cytokines, flaviviruses

## Abstract

The innate immune system is the host’s first line of defense against the invasion of pathogens including flavivirus. The programmed cell death controlled by genes plays an irreplaceable role in resisting pathogen invasion and preventing pathogen infection. However, the inflammatory cell death, which can trigger the overflow of a large number of pro-inflammatory cytokines and cell contents, will initiate a severe inflammatory response. In this review, we summarized the current understanding of the innate immune response, inflammatory cell death pathway and cytokine secretion regulation during Dengue virus, West Nile virus, Zika virus, Japanese encephalitis virus and other flavivirus infections. We also discussed the impact of these flavivirus and viral proteins on these biological processes. This not only provides a scientific basis for elucidating the pathogenesis of flavivirus, but also lays the foundation for the development of effective antiviral therapies.

## 1 Innate Immunity and Flaviviruses

### 1.1 Innate Immunity and Inflammasomes

As the first hurdle to protect the host from microbial invasion, the innate immune system can not only establish a rapid, broadly response to control infection, but also plays a key role in the establishment of an adaptive immune response, which can lead to pathogen-specific and durable immune memory (1). In order to quickly detect and resist a variety of pathogens, host cells have evolved many pattern recognition receptors (PRRs), including Toll-like receptors (TLRs), retinoic acid-inducible gene I (RIG-I)-like receptors (RLRs), and nucleotide-binding oligomerization domain (NOD)-like receptor family proteins (NLRs). When dealing with specific danger-associated molecular patterns (DAMPs) and pathogen-associated molecular patterns (PAMPs), members of the NLRs family are able to assemble large multiprotein complexes called inflammasomes (2). The NLRP3 (NLR family pyrin domain-containing 3) inflammasome is the most widely studied inflammasome that has been identified. NLRP3 is a cytoplasmic protein with three domains: the carboxy-terminal leucine-rich repeat sequence, the central nucleotide binding and oligomerization domain (NACHT) with ATPase activity, and the amino-termina pyrin domain (PYD) (3). Since the basal level of NLRP3 expression is usually not enough to activate the NLRP3 inflammasome. Therefore, a two-step process is required for priming and activation (4, 5). The priming step is induced by TLRs and cytokine receptors, such as the tumor necrosis factor receptor (TNFR) or IL-1 receptor (IL-1R), which recognize PAMPs or DAMPs and upregulate the transcription of NLRP3 and IL-1β. During the activation step, PAMPs and DAMPs promote NLRP3 inflammasome assembly. After assembly, the inflammasome induces the formation of membrane pores and the release of pro-inflammatory cytokines, which ultimately leads to a form of inflammatory cell death called pyroptosis (6, 7).

Innate immune response and inflammasome activation are recognized key obstacles in the process of virus invasion. On the other hand, the initiation of the innate immune response needs to be strictly regulated, since excessive activation could cause harmful tissue damage and systemic inflammation (8). Thence, the balance regulation between host’s innate immune responses and virus invasion is considered as a potential method for the treatment of viral infection. The balance should be well regulated to maintain antiviral function and avoid excessive inflammation.

### 1.2 Flaviviruses

The *Flavivirus* genus is composed of more than 70 positive-stranded RNA viruses transmitted by arthropods, especially mosquitoes and ticks. Flavivirus include pathogens of global concern such as Dengue virus (DENV), West Nile virus (WNV), Zika virus (ZIKV), Japanese encephalitis virus (JEV), Yellow Fever virus (YFV), Tick-borne encephalitis virus (TBEV) and Langat virus (LGTV). These viruses are arboviruses that can cause serious human infections, they pose a threat to global health and may cause serious outbreaks. These are demonstrated by the global distribution of DENV (9), the spread of ZIKV in South America (10), the outbreak of YFV in Brazil and Africa (11, 12), and the WNV outbreak in North America (13). The symptoms of flavivirus infection range from mild fever to joint pain to life-threatening hemorrhagic and encephalitis (14). Although vaccines against several of the viruses including DENV, JEV and YFV have been licensed, the outbreak is still happening. There is currently no clinical antiviral treatment for flavivirus infection, highlighting the challenge in implementing an effective vaccination program (15).

**Figure 1:**
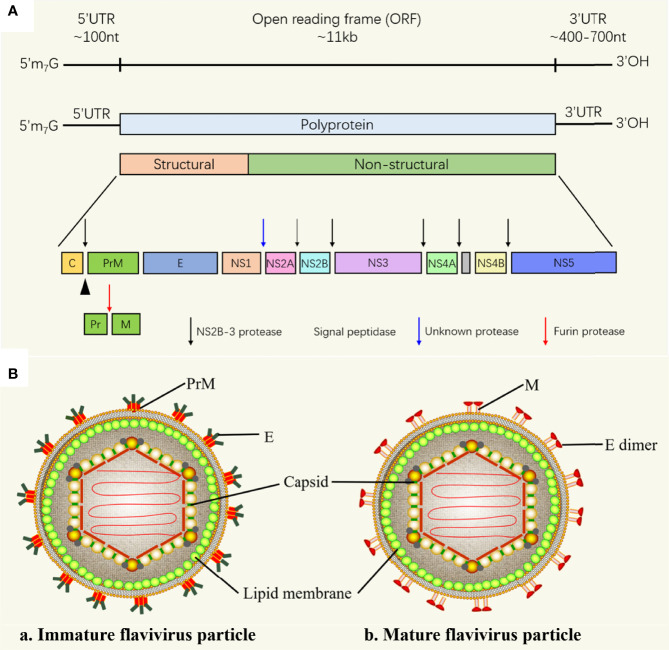
Flavivirus RNA genome and flavivirus particle. **(A)** Schematic representation of flavivirus genome. The polyprotein encoded by the genome is cleaved by the host and virus proteases to form 3 structural proteins: capsid (C), membrane (M) and envelope (E), and 7 nonstructural proteins (NS-1, 2A, 2B, 3, 4A, 4B and 5) (16). **(B)** Schematic diagram of the flavivirus particle model. (a) Immature flavivirus particles. The surface of immature virus particles is covered with 60 trimeric spikes, each of which is composed of a prM-E heterodimer (17). (b) Mature flavivirus particles. prM of is cleaved into M to form mature particles, there are 90 E homodimers and 90 M homodimers on the surface of each particle (18).

The approximately 11 kb flavivirus genome has only one open reading frame (ORF) flanked by a 5’-untranslated region (UTR) and a 3’-UTR, encoding 3 structural proteins [capsid (C); precursor of M (prM) and envelope (E)] and 7 non-structural (NS) proteins (NS1, NS2A/2B, NS3, NS4A/4B and NS5) (**Figure 1A**). Structural proteins mainly constitute virions, while NS proteins are involved in viral genome replication and viral particle assembly, and are involved in initiating host innate immunity (16). The nucleocapsid is composed of C protein wrapped RNA genome (17), nucleocapsid is surrounded by a lipid bilayer, M and E proteins are inserted into the lipid bilayer. In immature virus particles, M protein exists in the form of precursor protein (prM), the immature particle contains 60 trimeric spikes of prM-E heterodimers (**Figure 1B**). PrM is cleaved when the mature virus particle is formed (18), the mature flavivirus particle is composed of 90 E homodimers and 90 M homodimers on the surface, and the E protein is responsible for receptor binding, attachment, membrane fusion and viral entry.

**Figure 2:**
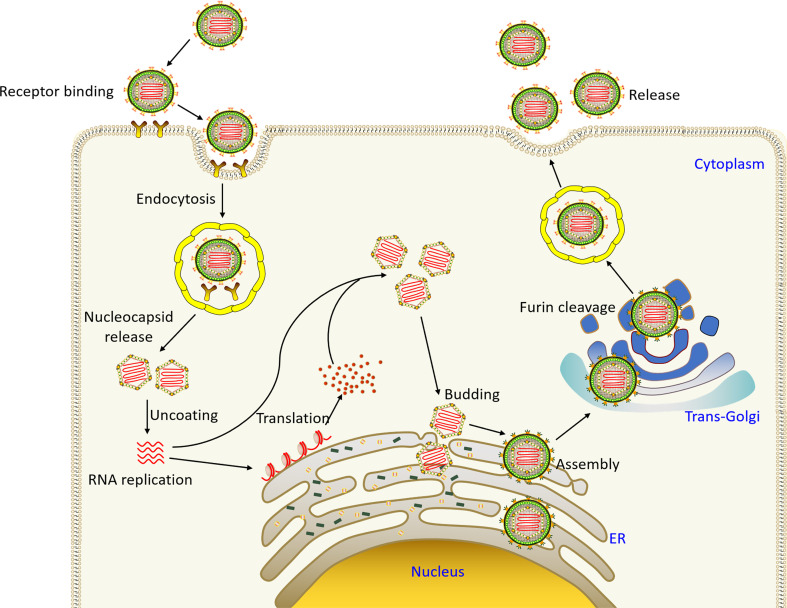
The flavivirus life cycle. Virus particles bind to receptors on the surface of the host cell membrane and enter the cell through endocytosis. The acid environment of endosomal vesicle induces the conformational changes of virus particles, and the fusion of virus and vesicle membrane leads to the release of virus particles. Subsequently, the viral genome is released into cytoplasm, and the positive-sense RNA is directly translated into a polyprotein, which is cleaved and processed by the virus and host proteases. Virus assembly occurs on the surface of the ER, and then non-infectious, immature virus particles carrying prM and E are transported to trans-Golgi network (TGN). The host protease furin then cleaves prM to M, producing mature infectious particles. Eventually mature virus particles are released by exocytosis (19).

In the process of infecting the host, flavivirus first attaches to the cell surface and enter host cell through endocytosis mediated by cell surface receptors (**Figure 2**). The acidic environment of endosome triggers the membrane fusion of the virus with the cell. Through membrane fusion, nucleocapsid is released to cytoplasm, capsid protein and the viral RNA are dissociated, the viral RNA genome begins to replicate, the viral proteins are expressed, and the viral particles begin to assemble. Initially, immature, non-infectious virus particles are formed in the endoplasmic reticulum, which cannot yet induce fusion with the cell membrane (19). Subsequently, immature virus particles are transported to Golgi apparatus, prM is cleaved to form mature M protein, E protein is rearranged, and mature infectious virus particles begin to form. Mature virus particles are released from host cell through cellular exocytosis.

Within this article, we discussed innate immune recognition, the activation of inflammatory cell death pathways, and the release of cytokines during flavivirus infection to promote resistance to viral infections; and described how flavivirus evade host innate immune response to promote viral infection.

## 2 Innate Immune Recognition of Flavivirus

**Figure 3:**
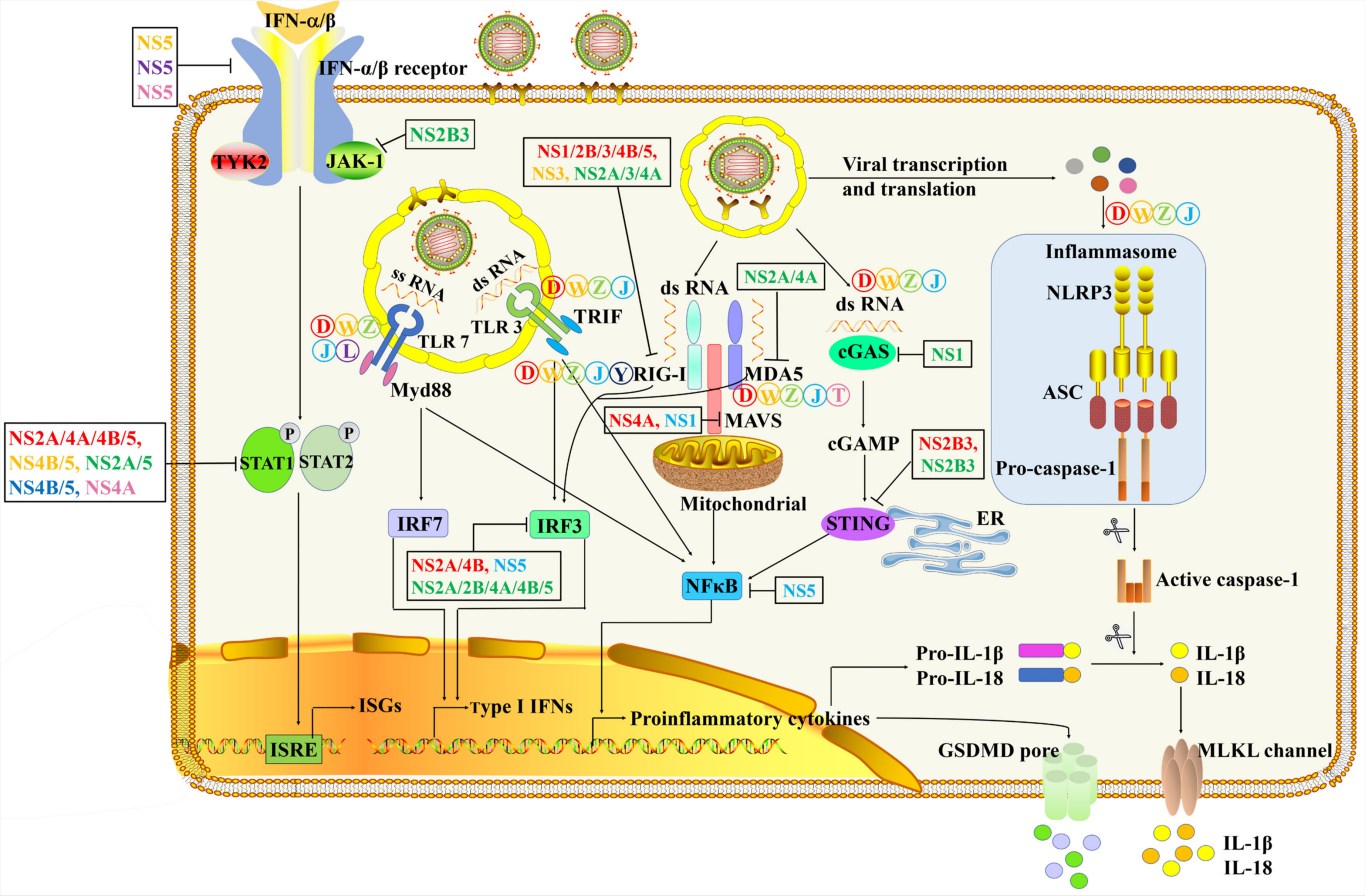
The innate immune pathways in the process of flavivirus infection and immune evasion. After flavivirus infection, TLR3 and TLR7 recognize double-stranded RNA (dsRNA) and single-stranded RNA (ssRNA), respectively. It then triggers a signal cascade downstream of the TLRs to induce the activation of NF-κB to produce pro-inflammatory cytokines and phosphorylation of IRF/7 to drive the production of type I IFN (24–32). The dsRNA RNA in the cytoplasm is recognized by RIG-I and MDA5 (33–36). The combination of RIG-I and MDA5 with MAVS leads to the activation of NF-κB and the phosphorylation of IRF3 (37–41). Severe flavivirus can also stimulate the formation of inflammasomes, leading to caspase-1 activation and release of the cytokines IL-1β and IL-18 (42–45).

The innate immune system is a strong barrier to prevent flavivirus infection. In a typical flavivirus infection process, viral RNA could be identified by a variety of PRRs, such as TLRs (20), RLRs (21, 22), cyclic GMP-AMP synthase (cGAS) and NLRs (23), which ultimately produce pro-inflammatory cytokines and induce antiviral status (**Figure 3** and **Table 1**). Typically, pro-inflammatory cytokines can trigger the infiltration of immune cells and eliminate infectious viral factors, which is beneficial to the host to a large extent. However, excessive pro-inflammatory cytokines can cause harmful tissue damage and systemic inflammation (53).

**Table 1:** Engaged innate immune sensors and notable cytokines released in response to specific flavivirus^a^.

Virus	PRRs		Notable cytokines	*In vivo* or *in vitro*	Cell lines	Strains	References
DENV	TLRs	TLR3	IFN-β	*in vitro*	HepG2	New Guinea C	(28)
	RLRs	TLR7	IFN-α/β	*in vitro*	pDCs	16681 strain, New Guinea C	(26)
	NLRs	RIG-I, MDA5	IFN-α/β	*in vitro*	DCs	16681 strain	(34)
	cGAS-STING	RIG-I, MDA5	IFN-β	*in vitro*	HUH-7	Singapore strain	(36)
		NLRP3	IL-1β/6, TNF-α	*in vivo*	HMEC-1	PL046 strain	(42)
		NLRP3	IL-1β	*in vitro*	A549, tdP-1	New Guinea C	(43)
		cGAS-STING	cGAMP	*in vitro*		PDK53 strain	(46)
WNV	TLRs	TLR3	IFN-α/β	*in vivo*		3000.0259 strain	(25)
	RLRs	TLR7	IL-6, IFN-β	*in vitro*	macrophages	CT-2741 strain	(20)
	NLRs	RIG-I, MDA5	IL-12, IL-23	*in vivo*	BMMs	CT-2741 strain	(21)
	cGAS-STING	RIG-I, MDA5	ATF4, SMAD4	*in vitro*		TX-02 strain	(33, 35)
		NLRP3	IFN-α/β	*in vivo*		TX-02, 3000.0259 strain	(23)
		cGAS-STING	IL-1β	*in vivo*		TX-02 strain	(47)
		TLR3	IFN-α/β, IRF3	*in vivo*		MHV68 strain	(24)
ZIKV	TLRs	TLR3	IFN-α/β	*in vivo*			(29)
	RLRs	TLR7/8	Viperin	*in vitro*	monocytes	MR766 strain	(31)
	NLRs	RIG-I, MDA5	IFN-β	*in vitro*	trophoblasts	FLR strain	(41)
	cGAS-STING	NLRP3	IL-1β	*in vitro*	THP-1	SZ01, z16006 strain	(44, 45)
		cGAS-STING	IFN-α/β	*in vitro*	fibroblasts	MR766 strain	(48)
JEV	TLRs	TLR3	TNF-α, IL-6, CCL-2	*in vitro*	BV-2	P3 strain	(27)
	RLRs	TLR7/8	TNF-α, IL-6, CCL-2	*in vivo*	BV-2	P3 strain	(32)
	NLRs	RIG-I	IFN-α/β, p38MAPK,	*in vitro*	Neuro2a	P3 strain	(27)
	cGAS-STING	RIG-I	NFκB	*in vitro*	MEFs		(37, 38)
		RIG-I, MDA5	IFN-α/β	*in vitro*	BV-2		(39)
		NLRP3	IL-1β/18	*in vitro*	MEFs		(49)
		cGAS-STING	TNF-α, IL-6	*in vitro*			(50)
				*in vitro*			
YFV	TLRs	N/A					
	RLRs	RIG-I	IFN-β, TNF-α, IL-6	*in vitro*	HUH-7	Asibi strain	(51)
	NLRs	N/A					
	cGAS-STING	N/A					
LGTV	TLRs	TLR7	IFN-α/β, IRF1	*in vivo*		TP21 strain	(30)
	RLRs	RIG-I, MDA5	IFN-α/β/λ	*in vivo*		Hypr strain	(52)
	NLRs	N/A					
	cGAS-STING	N/A					
TBEV	TLRs	N/A					
	RLRs	RIG-I, MDA	IRF3, RANTES	*in vitro*	T98G	WH2012	(40)
	NLRs	N/A					
	cGAS-STING	N/A					

a N/A, not applicable (data for a particular virus and receptor combination have not been reported).

### 2.1 TLRs- and RLRs-Mediated Flavivirus Recognition

Signal transduction mediated by TLRs and RLRs results in the secretion of type I interferon (IFN-I), which subsequently stimulates the expression of IFN-stimulating genes (ISGs) to establish an antiviral state (24–26). The TLRs and RLRs signaling cascade also induce the production of pro-inflammatory cytokines, such as interleukin 1β (IL-1β) and interleukin 18 (IL-18) (20).

TLR3 is a membrane-bound PRR located in the endosome, which could recognize dsRNA from DENV, WNV ZIKV and JEV (24, 25, 27–29). TLR3 activates TRIF in turn, and TRIF activates TRAF3/TBK-1/IKKϵ kinase complex through RIP-1. The complex then phosphorylates interferon regulatory factor 3/7 (IRF-3/7) causing their activation. The activated IRF3/7 then induce the transcription of IFN-I genes (24, 25, 28). RIP-1 can also activate IKKα/β kinase and then activate nuclear factor-κB (NFκB) to induce the expression of pro-inflammatory cytokines. TLR7, as another membrane-bound PRR, has also been shown to be involved in regulating the expression of IFN-I and pro-inflammatory cytokines in response to DENV, WNV, ZIKV, JEV and LGTV infection (20, 26, 30–32). After TLR7 recognizes ssRNA, it dimerizes and recruits the myeloid differentiation primary response 88 (MYD88) adaptor protein (20, 26). MYD88 then activates the transcription factors IRF7 and NF-κB, which stimulate the production of IFN-I and pro-inflammatory cytokines for host defense, respectively (30). Indeed, compared with wild-type mice, TLR7^-/-^ and Myd88^-/-^ mice have more severe disease after WNV infection, with reduced survival and increased virus transmission (20).

Melanoma differentiation-associated gene 5 (MDA5) is a cytoplasmic PRR belonging to RLRs family, it typically binds to double-stranded long RNA. Studies have found that it plays a vital role in responding to various flavivirus infections (33, 34). MDA5 is composed of two caspase recruitment domain (CARD) domains at the N-terminus and a DExD/H-box helicase domain at the C-terminus. When the C terminal domain binds to the viral ligand, MDA5 undergoes a conformational change, which allows the CARD domain to bind to mitochondrial antiviral signaling protein (MAVS), and ultimately induces the expression of interferon and pro-inflammatory cytokines (21, 35, 36). RIG-I, another member of the RLRs, can sense 5′-triphosphate-double-stranded RNA (22, 54). So far, RIG-I is participated in identifying almost every member of the *flavivirus* genus (51, 52). Similar to MDA5, after RIG-I recognizes cytoplasmic dsRNA, it interacts with MAVS located on mitochondria (55). This complex can activate both IRF-3/7 and NF-κB (37, 38), eventually increase the release of IFN-I and pro-inflammatory cytokines, and establish an antiviral state in the cell (39, 40). Accordingly, IFN-β induced by ZIKV decreased in RIG-I^–/–^ and MDA5^–/–^ cells, and completely abolished in MAVS^–/–^ cells, indicating RIG-I and MDA5 play an indispensable role in this process (41).

Interestingly, it’s recently reported that PARPs (poly-adenosine 5′-diphosphate (ADP)-ribose polymerases) family member PARP9 is a non-canonical sensor for RNA virus in dendritic cells (56). Additionally, DHX15 is identified as RNA sensor for RNA viruses and is required to control RNA virus-induced inflammation by activating NLRP6-mediated inflammasome (57, 58). Studies have identified PARP1 and PARP12 as strong inhibitors of ZIKV replication (59, 60). They inhibit virus replication by reducing intracellular ATP and NAD^+^ concentrations or mediating the degradation of NS1 and NS3 by the proteasome pathway, respectively. Whether the RNA sensors PARP9 and DHX15 have similar functions to control flavivirus infections requires more in-depth research.

### 2.2 cGAS-Mediated Flavivirus Recognition

Besides the RNA sensors, the DNA sensor cGAS has also been proven to detect and limit flavivirus (46, 47). cGAS is activated after binding to DNA in the cytoplasm, and cyclizes AMP and GMP in the cytoplasm to produce 2’, 3’-cGAMP. As the second signal, cGAMP continues to activate STING, which in turn promotes the expression of interferon and pro-inflammatory cytokines to exert antiviral effects. Studies have found that the disturbance of mitochondrial membrane induced by DENV leads to the leakage of mitochondrial DNA into the cytoplasm, which finally triggers the activation of the cGAS-STING signaling pathway and promotes downstream IFN gene expression (46). In addition, DTMUV infection of a variety of cell lines lacking STING found enhanced replication of DENV (61). ZIKV replication in STING^–/–^ human fibroblasts is enhanced, ZIKV also promotes infection by actively antagonizing STING in the cGAS pathway (48). Besides, JEV can also activate the cGAS-STING axis after infecting the mouse embryonic fibroblasts (MEFs) (50).

### 2.3 Type I IFN Response During Flavivirus Infection

Mammalian cells mainly sense flavivirus infections through PRRs (including TLRs, RLRs and cGAS), and then their downstream signaling pathways are activated, ultimately inducing the production of IFN-I. Then, the released IFN-I binds to the IFN-I receptors (IFNAR1/IFNAR2) to activate the JAK/STAT signaling cascade to initiate antiviral status.

First, IFN-I plays an important role in resisting DENV infection and generating an immune response (62). Other studies found that IFN-α/β plays a leading role in resisting WNV by limiting cell and tissue tropism infection (63), and TRIM6 helps establish IFN-I-induced antiviral response against WNV (64). In addition, IFN-I is essential to resist ZIKV, since IFN-I-mediated strong antiviral effects on ZIKV replication (>100-fold reduction) (65), while IFNAR-deficient mice are highly susceptible to ZIKV (66). Meanwhile, autophagy induced by ZIKV is conducive to activating host immunity through IFN-I signaling (67). The IFN-I response has also been shown to be a major obstacle to the viscerotropism and pathogenicity of JEV (68), such as IFN-I can limit hemorrhage-like disease after infection with JEV (69). Moreover, IFN-I is also essential to protect against LGTV and TBEV in mice at two different stages. The first stage inhibits virus replication and prevents its spread to the central nervous system (CNS) at the periphery. In the second stage, local IFN responses of the CNS can prevent the development of inflammation and encephalitis caused by the virus (52, 70, 71).

### 2.4 Flavivirus Infection Stimulates NLRP3 Inflammasome Activation

The innate immune response has significant effects on antiviral immunity, inflammatory signal transduction and cytokine production. Within pro-inflammatory cytokines, IL-1β and IL-18 are crucial factors that trigger inflammatory response. The inflammasome processes inactive pro-caspase-1 into active caspase-1, which cleaves pro-IL-1β/18 into mature IL-1β/18, leading to inflammation (72). The inflammasome sensors can identify PAMPs and DAMPs produced after pathogen infection (72). The elucidation of the NLRs family pyrin domain-containing 3 (NLRP3) is the most thorough among these sensors, and it is related to a variety of diseases, such as autoinflammatory diseases, obesity and colitis (73–79).

DENV can activate NLRP3-specific inflammasome in human patients and mice; specifically including human peripheral blood mononuclear cells (PBMCs), keratinocytes and platelets, as well as mouse bone marrow derived macrophages (BMDMs), endothelial and dendritic cells (80). After DENV infection, caspase-1 is activated by inflammasomes, which cleaves IL-1β and IL-18 to induce inflammatory response (42). In addition, DENV NS2A and NS2B proteins activate the NLRP3 inflammasome, which then increases the oligomerization of apoptosis-associated speck-like protein containing a CARD (ASC), and promotes caspase-1 activation and IL-1β secretion (43). Ramos et al. (23) found that NLRP3 signaling pathway is the most important way to trigger IL-1β production after WNV infection. ZIKV can stimulate human PBMCs, macrophages and mice BMDCs to secret IL-1β through NLRP3 inflammasome (44). Moreover, the combination of ZIKV NS5 protein and NLRP3 promotes the assemble of inflammasome complex to promote the secretion of IL-1β (45). However, overexpression of ZIKV NS3 protein can reduce the activation of caspase-1 and even degrade NLRP3, which ultimately inhibits IL-1β secretion (81). The efflux of K^+^ ion and the release of reactive oxygen species (ROS) mediated by JEV infection can also induce NLRP3 inflammasome activation (49).

In the host antiviral responses, the activation of NLRP3 inflammasome plays a crucial role (76, 77). However, excessive activation of NLRP3 inflammasome can also cause severe pathological damage. For example, the interaction of DENV M protein and NLRP3 causes over-activation of NLRP3 inflammasomes and excessive release of pro-inflammatory cytokine IL-1β, which ultimately leads to increased endothelial permeability and vascular leakage (82). Additionally, DENV E protein domain III (EIII) induces neutrophil death *in vitro* and *in vivo*, which also depends on NLRP3 and caspase-1 (83, 84). During ZIKV infection, acute kidney injury can also be induced by activating the NLRP3 inflammasome (85). The above findings confirm that proper activation of NLRP3 inflammasome is beneficial to the host, but abnormal activation may cause unfavorable results.

## 3 PANoptosis and Proinflammatory Cytokines During Flavivirus Infections

### 3.1 PANoptosis

**Figure 4:**
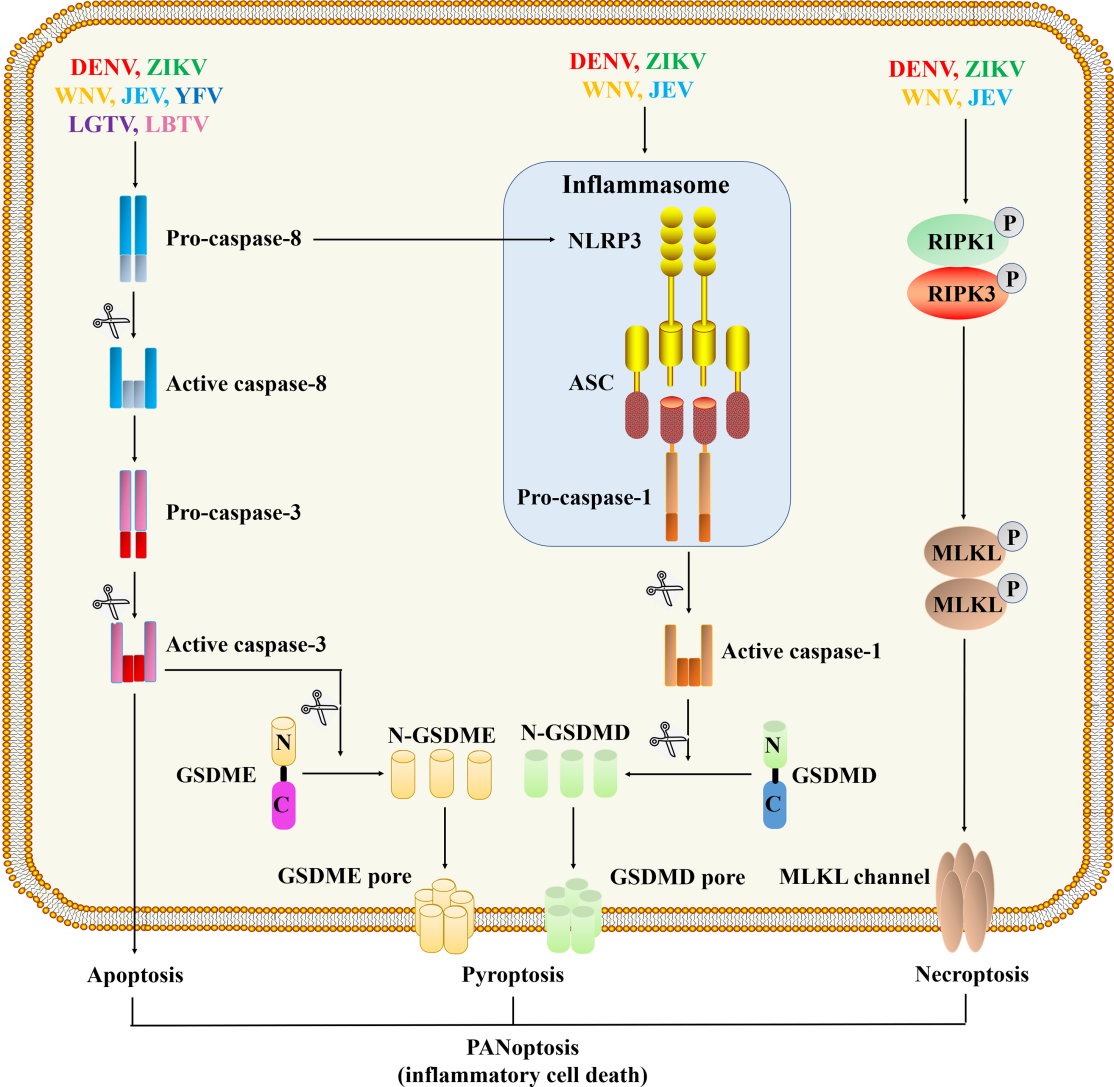
Programmed cell death pathways during flavivirus infection. The viral proteins of DENV (88–90), WNV (91), ZIKA (92, 93) and JEV (94) can act as cytoplasmic PAMPs to stimulate the assembly of inflammasomes, thereby activating caspase-1. Activated caspase-1 cleaves GSDMD, and then the N-terminal fragment of GSDMD oligomerizes in the membrane to form membrane pores and initiate pyroptosis. Flavivirus infections also initiate a signal cascade mediated by caspase-8, activated caspase-8 cleaves and activates caspase-3 to initiate apoptosis (95–98). WNV (68), ZIKA (99, 100) and JEV (101) infections can trigger necroptosis, which depends on the formation of RIPK1 and RIPK3 complexes and the activation of downstream MLKL proteins to form channels in the membrane.

Cell death plays a vital role in resisting pathogen invasion. On the other hand, inflammatory cell death can lead to the release of pro-inflammatory cytokines, cell contents, PAMPs and DAMPs, which will induce a severe inflammatory response (86, 87). The immune system has evolved a variety of mechanisms to limit microbial infections and regulate inflammation. The innate immune system recognizes microbial molecules that are conserved in many pathogens, and responds quickly by producing inflammatory mediators and activating programmed cell death pathways, including pyroptosis, apoptosis, and necroptosis. The activation of pattern recognition receptors, combined with inflammatory cytokine-induced signal transduction through receptors containing death domains, initiates a highly interrelated cell death process called PANoptosis (pyroptosis, apoptosis, necroptosis) (**Figure 4**).

### 3.2 PANoptosis During Flavivirus Infection

#### 3.2.1 Pyroptosis

Pyroptosis is a way of inflammatory cell death mediated by inflammasome and gasdermin (7). After receiving the pyroptosis signal, the inflammasome assembly causes the activation of inflammatory caspases (caspase-1/4/5/11), and the N-terminal fragment of gasdermin D (GSDMD) produced by the activated caspase is transported to the plasma membrane to form pores, resulting in the production of pro-inflammatory cytokines (2, 6, 7). Among them, the release of IL-1β and IL-18 caused by GSDMD cleavage is closely related to the activation of caspase-1 (2). Recent findings indicate that when infecting monocytes or macrophages, DENV activates the inflammasome and caspase-1, followed by the release of IL-1β and cellular contents, which ultimately induces pyroptosis (88–90). Another study showed that during DENV infection of macrophages, caspase-4 is located upstream of caspase-1 to regulate pyroptosis (102). In addition, both DENV EIII and NS1 proteins can induce pyroptosis through the inflammasome NLRP3, it further causes the typical manifestations of DHF (dengue hemorrhagic fever) such as vascular damage, liver dysfunction, thrombocytopenia, and hemorrhage (84, 103, 104). The pyroptosis induced by ZIKV infection directly affects the development of neural progenitor cells, which is closely related to the development of microcephaly (92, 93). ZIKV’s protease NS2B3 can directly cleave GSDMD in a caspase-independent manner to trigger cell pyroptosis, indicating a new mechanism for ZIKV to directly induce cell death and inflammation (105). Transcriptome analysis of JEV-infected peritoneal macrophages found that almost all PCD pathways, including pyroptosis, were activated after JEV infection (94) (**Table 1**).

#### 3.2.2 Necroptosis

The activation of necroptosis depends on the phosphorylation of MLKL (mixed-lineage kinase domain-like pseudokinase) regulated by RIPK3 (receptor-interacting serine/threonine protein kinase 3), which causes conformational changes and activation of MLKL. The activated MLKL translocate and form channels in the plasma membrane (106). Increased expression of RIPK1, RIPK3 and MLKL proteins was detected in ZIKV-infected astrocytes, indicating that ZIKV induced necroptosis; after inhibiting necroptosis, virus replication increased significantly, indicating that necroptosis has a resistance to virus replication (99). In addition, retinopathy caused by ZIKV is associated with inflammation mediated by necroptosis (100). Bian et al. (101) demonstrated that necroptosis is related to neuronal loss during JEV infection, providing evidence for necroptosis to participate in the pathogenesis of JEV infection. Transcriptomics analysis of JEV-infected macrophages revealed that the necroptotic pathway was activated, which was confirmed by the immunofluorescent staining with specific markers (94). Similarly, transcriptomics has also found evidence of differential expression of markers of pyroptosis and necroptosis during WNV and CHIKV neuroinvasive diseases, but more research is needed to explain the role of inflammatory cell death in viral neuroinvasive diseases (91). Alternatively, necroptosis can also be activated by the sensor Z-DNA-binding protein 1 (ZBP1), which is an ISGs containing the RHIM domain to recruit and activate RIPK3-induced MLKL phosphorylation, leading to cell death. It has been reported that ZBP1-mediated cell death is involved in various viral infections including WNV (107) and ZIKV (108).

#### 3.2.3 Apoptosis

Pyroptosis and necroptosis-mediated lytic forms of cell death are driven by GSDMD pores or MLKL channels, respectively. They release inflammatory factors and other cytokines to alert nearby cells of danger and recruit innate and adaptive immune cells (86, 87). Apoptosis was originally thought to be a way of cell death that does not cause inflammation. It breaks down cells by forming apoptotic bodies that wrap the cellular contents, which is finally cleared by phagocytes (109). But new research shows that apoptosis is not always immune-silent because of the signal crossing between it and the lytic cell death pathways (109). Apoptosis is driven by the initiator caspase-8, -9 and -10 cleavage executor caspase-3 and -7. Apoptosis is induced by the “initiator” caspase-8, -9 and -10 cleavage downstream “executor” caspase-3 and -7. According to reports, caspase-3 and caspase-8 can cleave and activate GSDME (gasdermin E) or GSDMD, respectively, leading to inflammatory cell death (109, 110).

DENV can induce apoptosis in a variety of cells, such as mouse neuroblastoma cells [Neuro 2a (111)), liver cancer cells (HepG2 (112), Hep3B (113)], endothelial cells (114) and PBMCs (115), and human monocyte-derived dendritic cells (Mo-DC) (116). Among them, DENV2 induces vascular endothelial cell apoptosis through FasL/Fas and XIAP-related factor 1 (XAF1)-dependent pathways, which is related to vascular endothelial dysfunction in the pathogenesis of DHF (117). And sphingosine kinase 2 (SPHK2) plays a pro-apoptotic effect in DENV-infected liver cells, which is associated with liver dysfunction (118). Within 2 hours of being infected with DENV or ZIKV, apoptosis of *Aedes aegypti* midgut epithelial cells was rapidly induced to prevent virus proliferation (119), similar results were observed in the midgut cells of WNV-infected *C. p. pipiens (* 120). In addition, DENV C protein induces apoptosis by localizing to the nucleus and interacting with Fas death domain associated protein xx (DAXX) (121). The DENV-M ectodomain can activate the mitochondrial-dependent apoptotic pathway after being transported from the Golgi apparatus to the plasma membrane, the M ectodomain of JEV, WNV and YFV also has pro-apoptotic properties (122, 123). In addition, DENV-EIII inhibits megakaryopoiesis by activating the apoptosis of its progenitors, which is associated with thrombocytopenia that is frequently observed in patients with dengue fever (124). However, DENV-NS1 can interact with the key autophagy gene Beclin-1 to inhibit the degradation of Beclin-1, and ultimately promote autophagy and prevent cell apoptosis (125). The proteases NS2B3 and NS3 can trigger apoptosis *via* the caspase-8 or NF-κB pathway (95, 126). Another study found that DENV-NS2B3 caused endothelial cell apoptosis by activating NF-κB pathway, indicating that NS2B3 is involved in the pathogenesis of DHF (127). Similarly, NS3 of JEV and WNV can also induce apoptosis by activating caspase-3 or caspase-8, leading to extensive damage to the nervous system (95, 96).

WNV induces cell death of various cells with the participation of extrinsic and the intrinsic apoptotic pathways (128, 129). Cellular microRNA Hs_154 was significantly up-regulated after WNV infection, and subsequently caused apoptosis by targeting anti-apoptotic protein (130). WNV C protein interacts with importin-α and triggers phosphorylation of protein kinase C to induce apoptosis (131). On the contrary, WNV-C can activate PI3K/AKT signaling pathway to inhibit the activation of caspase-3 and 8 (97), and other flavivirus capsid proteins can also protect cells from apoptosis by activating Akt (132). WNV-NS2A is also involved in apoptosis and pathogenesis, because after NS2A mutation (converting alanine 30 to proline (A30P)), the quantity of TUNEL (terminal deoxynucleotidyl transferase dUTP nick end labeling) positive cells is greatly decreased (133).

ZIKV infection triggers apoptosis of human neural progenitor cells (134), as evidenced by the activation of caspase 3, 7, 8 and 9, leading to cortical thinning and microcephaly (98). Inhibition of tumor suppressor protein p53 prevents ZIKV-mediated apoptosis of neural progenitor cells, confirming p53 is involved in ZIKV-induced apoptosis (135). The ZIKV-C induces ribosomal stress and apoptosis, and the C protein of DENV has the same function (136). ZIKV-M oligopeptide ZAMP can induce apoptosis by activating caspase-3/7 (137). Interestingly, the subgenomic flaviviral RNA (sfRNA) of ZIKV promotes the spread of ZIKV by inhibiting cell apoptosis in mosquito tissues (138).

JEV can induce apoptosis through multiple signal pathways. It can activate IRE1/JNK pathway of endoplasmic reticulum stress (ERS) to induce apoptosis (139), it can also inhibit the STAT3-Foxo-Bcl-6/p21 pathway to trigger apoptosis (140). What’s more, JEV-NS4B induces apoptosis through the PERK-ATF4-CHOP pathway in response to ER stress (141). Lee et al. showed that the PI3K/Akt pathway triggered by JEV and DENV-2 has an anti-apoptotic effect to protect infected cells from early apoptosis (142). In addition, LGTV-E also induces apoptosis (143), and LGTV-NS3 is a multifunctional protein that binds to caspase-8 and induces apoptosis (144). Activation of the apoptotic pathway can also be observed in TBEV-infected DCs (145). Collectively, these findings indicate that flavivirus can regulate cell apoptosis (**Table 1**).

### 3.3 Excessive Pro-Inflammatory Cytokines and Diseases

Excessive activation of the inflammatory cell death can cause severe inflammation and tissue damage (86). For instance, in patients with dengue fever, the severity of the disease is connected with high levels of IL-1RA and CXCL10 in the plasma (146). The detection of severe cases of dengue fever found that the expression of pro-inflammatory cytokines (IL-1, TNFα), anti-inflammatory cytokines IL-10 and chemokines (IL-8, CXCL10) were significantly up-regulated, and this cytokine storm was associated with plasma leakage and hemorrhage (147). The recognition of DENV NS1 by TLR4 can also lead to the pro-inflammatory cytokines production, which contributes to vascular damage (148). In addition, some pro-inflammatory cytokines and IFN-stimulated chemokines are closely related to the severity of ZIKV. Studies have found that ZIKV patients with moderate symptoms and viremia have higher levels of IL-8, IL-1RA, CXCL10 and CCL2 compared with patients with mild symptoms or no viremia (149). This means that immunopathology is an important part of flavivirus pathogenesis, and we need further research to fully clarify the pathways and functional consequences of these pro-inflammatory cytokines released during flavivirus infection.

## 4 Flavivirus Immune Evasion

The innate immune response uses PRRs to identify pathogens and triggers inflammatory response and programmed cell death to prevent virus invasion and promote its clearance. On the other hand, flavivirus have evolved the ability to limit the innate immune responses to promote viral replication. Among them, the most in-depth research on the immune evasion mechanism of flavivirus is the regulation of type I interferon signals (**Figure 3**).

DENV NS1 protein interacts with ApoA1, a key component of HDL (high-density lipoprotein), to change the sensitivity of the membrane to viral infections, and ultimately evade the immune response (150). ZIKV-NS1 can inhibit IFN-α response mediated by CD303 (151), Xia et al. (152) found that ZIKV-NS1 inhibits RIG-I-mediated activation of IFN-β promoter, the same author proved that ZIKV NS2A/B, NS4A/B and NS5 could inhibit IFN-β by reducing the phosphorylation of IRF3 at Ser-396. New research shows that cGAS is cleaved by caspase-1 downstream of the NLRP3 inflammasome activated by ZIKV-NS1 (153). Furthermore, ZIKV NS1 and NS4B interact with TBK1 to prevent TBK1 oligomerization, phosphorylation and its mediated activation of IFN-I (154). The NS1 of JEV could inhibit the production of IFN-I by targeting MAVS (155).

In addition, DENV NS2A, NS4A and NS4B can suppress the transcription of ISRE promoter and ISGs by blocking the phosphorylation of STAT1/STAT2 and hinder their nuclear localization to inhibit the IFN response (156), and the NS2A of ZIKV and the NS4B of WNV and YFV have similar functions (157–159). NS2A and NS4B from multiple DENV serotypes can also inhibit IFN production by targeting IRF3 and TBK1, but only NS4A from serotype-1 can inhibit TBK1 (160). ZIKV NS2A and NS4A proteins antagonize the production of IFN-β mediated by MDA5/RIG-I (161). The conserved phosphomimetic motif in NS3 of DENV, WNV and ZIKV competes with RIG-I to bind 14-3-3ϵ, and finally prevents RIG-I from translocating to mitochondria (162). The expression of DENV NS2B can suppress the cGAS/STING-dependent IFN-β promoter activity and down-regulate the level of cGAS protein (163), DENV NS2B3 protease inhibits the production of type I IFN by cleaving STING (61, 164). Similar to DENV NS2B3, ZIKV NS2B3 also cleaves STING to inhibit IFN production (48). Furthermore, ZIKV NS2B3 degrades JAK1 in a proteasome-dependent manner, which ultimately leads to the down-regulation of IFN-mediated ISGs expression (165). According to reports, DENV NS4A binds to MAVS and blocks its interaction with RIG-I and downstream innate immune signals (166). In addition, TBEV antagonizes IRF-1 and IFN-I signaling to suppress dendritic cells function (167), and TBEV-NS4A can inhibit the phosphorylation of STAT1 and STAT2 to block type I and II IFN signaling (168). DENV NS4B can also trigger mitochondria elongation, causing altered MAMs (mitochondria-associated membranes) and reducing IFN production-possibly by preventing the recruitment of activated RIG-I to MAMs (169).

The NS5 of DENV and other flavivirus (WNV, ZIKV, YFV, JEV, TBEV, LGTV) can inhibit IFN signaling by targeting different steps and participants of the IFN-I signaling pathway (170–173). The NS5 protein of DENV and ZIKV binds to STAT2 and degrades STAT2 *via* the proteasome pathway, thereby inhibiting IFN-α signal transduction (170, 174–177), while YFV NS5 interacts with STAT2 and inhibits downstream ISRE activation (178). The DENV NS5 2′-O-methylation of 5′ also protects the virus from detection by RIG-I (179). Further experiments showed that ZIKV NS5 localizes to the nucleus and inhibits IRF3-mediated IFN-I transcriptional activation, and independent of its effect on STAT2 degradation (180). Additionally, the interaction between ZIKV NS5 and IKKϵ leads to a decrease in IKKϵ protein level and phosphorylation, thus blocking the activation of IRF3 (181). Meanwhile, WNV-NS5 has been proven to effectively prevent STAT1 phosphorylation and translocation to the nucleus (158, 182); JEV NS5 protein blocks IFN-I signaling and antiviral response by inhibiting the activation of transcription factors IRF3 and NF-κB (183). Moreover, the NS5 protein of TBEV, LGTV and WNV can interfere with the maturation of the IFNAR1 receptor and thus affect the IFN-I signaling pathway (184–186). In addition, DENV’s sfRNA can interact with TRIM25 to prevent its activation and interaction with RIG-I (187). ZIKV sfRNA can inhibit IFN-β promoter activation mediated by RIG-I or MDA5 (188), while JEV sfRNA reduces IRF3 phosphorylation and nuclear translocation, as well as downstream IFN-β expression (189).

## 5 Concluding Remarks

We discussed the perception and recognition of various members of flavivirus by the innate immune system, and the inflammatory cell death pathways initiated by the host in response to flavivirus infections. Appropriate cell death and inflammatory cytokines release are beneficial for host to resist virus invasion. On the contrary, excessive cell death and inflammation can cause harmful cytokine storms and tissue damage. Therefore, the innate immune response and cell death induced by flavivirus need to be strictly regulated to avoid excessive inflammatory response while maintaining antiviral function.

The reduction of innate antiviral defense ability and the excessive production of inflammatory cytokines may be some of the driving characteristics of flavivirus-mediated diseases. Therefore, we should design treatment strategies based on the mechanisms by which different flavivirus regulate innate immune responses, and evaluate the clinical efficacy of targeted innate immune pathways. This article will help us deeply understand the recognition and response of the innate immune system after flavivirus infection, as well as the regulation of different cell death pathways, and lay the foundation for further development of antiviral strategies.

## Author Contributions

YP and WC contributed ideas for the review and wrote the manuscript and produced the figures. AC, MW, ZY, and RJ edited and revised the manuscript. All authors contributed to the article and approved the submitted version.

## Funding

This work was supported by the National Natural Science Foundation of China (32172833), and the Program Sichuan Veterinary Medicine and Drug Innovation Group of China Agricultural Research System (SCCXTD-2021-18), and by China Agriculture Research System of MOF and MARA.

## Conflict of Interest

The authors declare that the research was conducted in the absence of any commercial or financial relationships that could be construed as a potential conflict of interest.

## Publisher’s Note

All claims expressed in this article are solely those of the authors and do not necessarily represent those of their affiliated organizations, or those of the publisher, the editors and the reviewers. Any product that may be evaluated in this article, or claim that may be made by its manufacturer, is not guaranteed or endorsed by the publisher.

## Abbreviations

PRRs, pattern recognition receptors; TLRs, Toll-like receptors; RLRs, retinoic acid-inducible gene I (RIG-I)-like receptors; NLRs, nucleotide-binding oligomerization domain (NOD)-like receptor family proteins; PAMPs, pathogen-associated molecular patterns; DAMPs, danger-associated molecular patterns; DENV, Dengue virus; WNV, West Nile virus; ZIKV, Zika virus; JEV, Japanese encephalitis virus; YFV, Yellow Fever virus; TBEV, Tick-borne encephalitis virus; LGTV, Langat virus; cGAS, cyclic GMP-AMP synthase; IFN, interferon; ISG, IFN-stimulating genes; IL-1β/18, interleukin 1β/18; IRF-3/7, interferon regulatory factor 3/7; NFκB, nuclear factor-κB; MYD88, myeloid differentiation primary response 88; MDA5, melanoma differentiation-associated gene 5; CARD, caspase recruitment domain; MAVS, mitochondrial antiviral signaling protein; CNS, central nervous system; NLRP3, NLR family pyrin domain-containing 3; PCD, programmed cell death; GSDMD/GSDME, gasdermin D/E; MLKL, mixed-lineage kinase domain-like pseudokinase; RIPK, receptor-interacting serine/threonine protein kinase.
